# Weather, climate, and climate change research to protect human health in sub-Saharan Africa and South Asia

**DOI:** 10.1080/16549716.2021.1984014

**Published:** 2022-04-04

**Authors:** Maria Nilsson, Ali Sie, Kanyiva Muindi, Aditi Bunker, Vijendra Ingole, Kristie L Ebi

**Affiliations:** a Department of Epidemiology and Global Health, Umeå University, Umeå, Sweden; b Nouna Health Research Centre, National Institute of Public Health, Burkina Faso; c African Population Health Research Center, Nairobi, Kenya; d Heidelberg Institute of Global Health (HIGH), Faculty of Medicine and University Hospital, Heidelberg University, Heidelberg, Germany; e Vadu HDSS, KEM Hospital Research Centre, Pune, India; f Center for Health and the Global Environment, University of Washington Seattle, Seattle, WA, USA

**Keywords:** Climate change, health impacts, demographic surveillance sites, Sub-Saharan Africa, South Asia

## Abstract

Weather, climate, and climate change are affecting human health, with scientific evidence increasing substantially over the past two decades, but with very limited research from low- and middle-income countries. The health effects of climate change occur mainly because of the consequences of rising temperatures, rising sea levels, and an increase in extreme weather events. These exposures interact with demographic, socio-economic, and environmental factors, as well as access to and the quality of health care, to affect the magnitude and pattern of risks. Health risks are unevenly distributed around the world, and within countries and across population groups. Existing health challenges and inequalities are likely to be exacerbated by climate change. This narrative review provides an overview of the health impacts of weather, climate, and climate change, particularly on vulnerable regions and populations in sub-Saharan Africa and South Asia, and discusses the importance of protecting human health in a changing climate; such measures are critical to reducing poverty and inequality at all scales. Three case summaries from the INDEPTH Health and Demographic Surveillance Systems highlight examples of research that quantified associations between weather and health outcomes. These and comparable surveillance systems can provide critical knowledge to increase resilience and decrease inequalities in an increasingly warming world.

## Background

Healthy populations are a prerequisite to facilitate the transitions needed to achieve a resilient and sustainable development. Weather, climate, and climate change are affecting human health, with the scientific evidence increasing substantially over the past decades [1]. Measures to protect human health are critical to reducing poverty and inequality at all scales. Future health risks from climate change will significantly affect the vulnerability of individuals, communities, and health systems. It will have a crucial bearing on whether and how the Sustainable Development Goals will be met by 2030.

Knowledge about how human health and wellbeing are affected and the range of policies and measures that need to be implemented to protect health has local to global dimensions. This understanding comes not just from health research and implementation, but also from upstream drivers of health, including ecosystems, agriculture, and water. Climate change is altering the functioning of these systems in ways that affect human health [2]. Therefore, health is not just the outcome of global environmental change, but also a driving force and facilitator of social and economic development.

The health effects of climate change occur mainly from the consequences of rising temperatures, rising sea levels, and an increase in extreme weather events [3]. These challenges interact with demographic, socio-economic, and environmental factors, affecting the size and the pattern of risks. Health can be directly affected by extreme weather events such as heat waves, floods, and droughts or indirectly through exposure to air pollution, poorer water quality and lack of access to clean water, deterioration in food supply and food security, and the establishment of new disease-carrying vectors and a changing distribution pattern of infectious diseases [3]. Examples of the health effects are injuries, illnesses, disabilities, and deaths associated with extreme weather events, heat-related illness and mortality, cardiovascular and lung disease, deterioration in asthma and other respiratory diseases, allergies, undernutrition, food, water, and vector-borne infectious diseases, and mental illness [3].

Differences in vulnerability between different parts of the world depend on national, regional, and local capacity to manage and prepare for the effects of climate change, which in turn depends on economics, social stability, and access to and prioritization of resources for health systems [2,4–6]. Further, risks are unevenly distributed around the world and are affected by social and economic development as well as access to and the quality of health care. Existing health challenges and inequalities are likely to increase between different regions with climate change, within countries and across population groups. Particularly vulnerable groups include the elderly, children, pregnant women, as well as those who suffer from diseases such as cardiovascular disease, diabetes, lung disease, and mental illness [3].

Extreme events such as drought and floods may lead to food insecurity, which in turn may interconnect with human population movement in the coming decades. Different climate change effects may lead to large, forced migration. Estimates vary of the number of people that may be impacted, but the UN estimates that about half of the world’s population by 2030 will live in areas of high water-stress and that increasing water shortages in already arid and semi-arid areas could lead to the displacement of between 24 and 700 million people [7]. The 2017 Lancet Countdown report estimated that within the next 90 years, over a billion people could be forced to migrate if measures are not taken to prevent polar ice from melting [8].

This narrative review gives an overview of the health impacts of weather, climate, and climate change, on vulnerable regions and populations in sub-Saharan Africa and South Asia, and discusses research needs and the potential role of research infrastructure in building local knowledge and capacity in LMICs. Three case summaries from the INDEPTH Health and Demographic Surveillance Systems highlight research that quantified associations between weather and health outcomes. These and comparable surveys can provide critical knowledge to increase resilience and decrease inequalities in an increasingly warming world.

## Weather, climate, climate change, and health effects in Sub-Saharan Africa and South Asia

The health risks from climate change are increasing in these regions mainly due to increasing temperatures and sea levels, more extreme weather, changes in precipitation patterns and drought-related water and food shortage [9]. Climate change may affect health by changing the frequency and severity of health issues that are already impacted by adverse climatic or weather events in the regions and by creating new threats in new places.

The Lancet Commission report on climate change and health suggested that the highest regional burden on health associated with climate change was most likely in sub-Saharan Africa [4]. Already being burdened by climate-sensitive diseases whilst having low preparedness, adaptive and response capacity at the institutional and community levels, sub-Saharan Africa is particularly vulnerable to the health impacts of climate change. However, research is limited. Projections indicate that South Asia, which is already very vulnerable to climate change, during the current century will experience some of the highest increases in annual average temperatures [10]. South Asia’s topography with high mountains and plains, long coastlines and low-lying islands increases the region's vulnerability to extreme weather events.

Heat exhaustion and heat stroke threaten people’s lives and may aggravate conditions for people with pre-existing cardiovascular and respiratory diseases. In 2015 around 3500 people died in a heatwave that affected parts of India and Pakistan [11,12]. Changes in temperature and heat waves have been reported in Africa and a statistically significant increasing trend of warming and heat wave occurrence was found in all African regions [13]. The association between heat waves and high temperatures with morbidity and mortality is well known, but despite the existing understanding of how it can hit extra hard on already vulnerable regions and populations, studies from sub-Saharan Africa are few.

Increasing temperature and more heavy and frequent rainfall have altered the distribution of infectious diseases such as dengue, an increasing health threat and economic burden in South Asia [14,15]. Improved suitability for habitats and transmission of mosquito-borne diseases may lead to future increases in malaria; however, it is not yet clear if the distribution of the vector and the pathogen are changing due to climate change. An estimated 94% of the global malaria burden occurred in Africa in 2019 [16].

Drought and saltwater intrusion from sea-level rise may worsen food insecurity due to loss of cultivable land in the South- and Southeast Asian regions, already struggling with high levels of undernutrition among children. Crop failures have been caused by altered and more unpredictable patterns of rainfall and fluctuations in temperature [17,18]. In Africa, food availability might be impacted by high temperatures and increased drought, changes in the amount and timing of rainfall, and increased flooding. There is consensus on the significant negative impact of climate change on all aspects of food security in Africa [9]. Flash floods in Bangladesh, India, and Nepal in recent years have claimed around 1500 lives and in Nepal led to food insecurity for almost 500,000 people [19].

By 2050, a mean sea-level rise is projected of up to 38 cm along India’s coast [9]. And in the Bengal Bay region consisting of the Bangladesh and eastern India coasts, cyclonic events have increased substantially due to increasing sea surface temperature and sea-level rise [20]. Such events may result in loss of livelihoods, displacement of people, and a number of negative health impacts for already vulnerable and marginalised populations.

Populations in LMICs may generally be more vulnerable to shifts in climate and extreme weather due to underlying vulnerabilities and combined pressures of poverty, undernutrition, weak institutions, and overburdened public health infrastructure. Poverty is widespread in sub-Saharan Africa, increasing sensitivity and reducing the capacity for managing climate change impacts on people. The number of people living in poverty grew with more than 125 million between 1990 and 2015 according to the World Bank [21]. Out of the world’s 28 poorest countries 27 are found in sub-Saharan Africa [22].

Migrants and the unhoused may be particularly vulnerable [23]. Around one billion people in the world live in slum areas, and according to trends for urbanization, the number is expected to increase in the future. In overcrowded slum communities in resource poor countries, adverse health effects are particularly severe during i) periods of extreme heat, with houses/shelters/roofs often built of metal, with lack of clean water and sanitation, and access to health care, and ii) climate-related hazards such as heavy rainfall and flooding impacting water quality, housing, and sanitation [24–26].

The following three case summaries highlight examples of research conducted in Sub Saharan Africa and South Asia. These case studies present associations between weather/climate and health and mortality outcomes in vulnerable regions with vulnerable populations.

## Case study 1: slum communities in Nairobi, Kenya

Climate change is expected to contribute to excess cardiovascular and respiratory mortality through extreme heat, particularly among older persons [2,27]. Further, climate-induced rainfall variability is expected to impact water quality and housing through flooding events, as well as affect food security [2,27].

The health risks of climate variability and change have greater impacts in areas with poor health infrastructure. For example, in most countries on the African continent, slums account for over 50% of the urban population [28]. With poor housing, crowding, and limited access to amenities and services such as proper healthcare, water, and sanitation, these areas remain very vulnerable to climate change-related events such as flooding [29,30]. Egondi et al., 2012 assessed the relationship between daily weather and mortality in two slum communities in Nairobi, Kenya: Korogocho to the north and Viwandani to the east of the city [31]. These communities form the demographic surveillance area for the Nairobi Urban Demographic and Health Surveillance System (NUHDSS).

### Methods

Mortality data were collected between 2003 and 2008. The cause of death was determined through Verbal Autopsy [32–34]. Time-series data analysis was conducted, adopting a quasi-Poisson model. With an elevation of about 1,700 meters Nairobi’s climate is cool throughout the year. Cold and heat thresholds for all-cause mortality were observed at 17°C and 20°C; lags up to 13 days were analysed. The effect of rainfall on mortality was assessed over 30 days. Analyses were stratified by age groups, gender, and cause of death.

### Results

**Figure 1.**
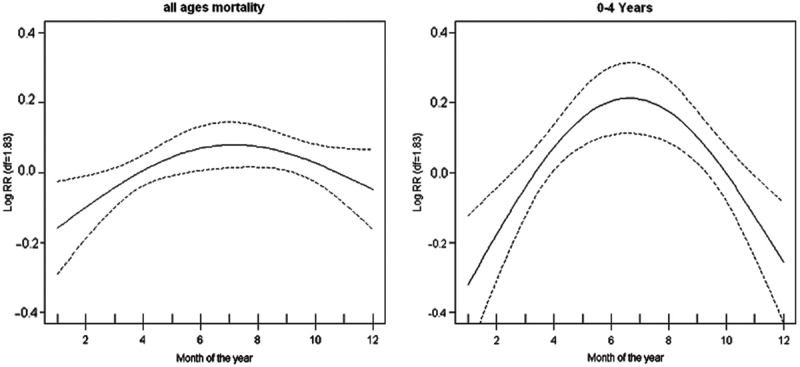
Annual seasonal variation plots for all-age and under-five mortality in Korogocho and Viwandani, Nairobi, Kenya. The vertical axes show the log (relative risk) and the horizontal axes show the month starting with January.Confidence intervals(95%) are shown as dotted lines (Source: Egondi et al, 2012).

There were 2,512 non-accidental deaths, with 33.7% among children younger than 5 years and 13.9% among adult 50 + . Mortality peaked during the rainy and cold months, with pneumonia and other respiratory illnesses likely causes of death. Figure 1 shows seasonal variation in all-cause mortality, with an inverted U-shaped relationship between mortality and month, more marked in children. The mortality peaks coincide with high rainfall months and the winter period.

### Conclusion

Respiratory deaths may be partially attributed to elevated household air pollution due to the use of charcoal or other biomass fuels for cooking and space heating [35]. Among older people, elevated temperatures may precipitate cardiovascular events due to poor thermoregulation [36]. Interventions for adaptation to climate change must consider slum housing given the large numbers of vulnerable people living in slums.

## Case study 2: Kossi province, rural Burkina Faso

Bunker et al. investigated the effect of heat exposure on premature death or years of life lost (YLL), from non-communicable diseases (NCD) in the Nouna HDSS in rural Burkina Faso between 2000 and 2010 [37].

### Methods

The study included 18,367 YLL from 790 NCD deaths over 11 years. Time-series regression was applied, assuming a quasi-Poisson distribution of daily YLL. Excess mean daily YLL from NCDs were based on the relative risk of maximum daily temperature, including lagged effects to 14 days.

### Results

Mean daily NCD-YLL was 4.6, 2.4, and 2.1 person-years for all-ages, males, and females, respectively. A moderate rise in the four-day cumulative maximum daily temperature resulted in a loss of 4.44 YLL for all ages and almost doubled to 7.39 at the 95th percentile of temperature. Premature deaths were highest on the day of heat exposure (lag 0), leading to 0.81 YLL. At lag 0, excess YLL were higher for males than females.

### Conclusions

This study investigates the influence of two understudied and important public health issues currently affecting a subsistence farming community in the Sahel: increasing ambient heat from climate change and the rising prevalence of NCDs. A significant rise in premature death from NCDs was observed with exposure to moderate and extreme heat in the Nouna HDSS. These findings highlight the need for adaptation and mitigation measures to tackle increasing ambient heat from climate change and to reduce the associated growth of NCDs. Morbidity data were not available. HDSS sites should expand data collection to include morbidity or years lived with disease (YLD) to fully quantify the burden of heat exposure on human health. Continued support of HDSS sites is required for maintaining and collecting accurate weather data, including temperature, humidity, and rainfall, using local weather stations, instead of modelled or proxy weather station data.

## Case study 3: heat and mortality in rural Pune, India

Expected increases in temperature in India and the frequency and intensity of extreme heat events due to climate change have become serious threats to human health [3]. The summer of 2016 was the hottest on record in India, where the highest ever-recorded temperature was 51^°^C. Due to data inaccessibility, very few research studies quantified the effects of weather on mortality in rural settings [38]. Ingole et al. (39) quantified weather-related mortality in the rural population in the Vadu Health and Demographic Surveillance System (HDSS) area that covers 22 villages from Pune district of Maharashtra, India, providing primary health care for about 130,000 individuals [39,40].

### Methods

This study included daily mortality data from 2003 to 2013 and applied time-series regression modelling controlled for seasonality and long-term trends [41]. Both short- and long-term lag periods were studied to assess heat and cold effects among the Vadu HDSS population.

### Results

Hot and cold temperatures were associated with daily mortality. Short-term associations were observed for high and low temperature, with up to 2 weeks lag [42]. The associations were stronger among children, women, and older adults [38,42]. Non-infectious disease mortality was higher on hot days (>39^°^C); and mortality from infectious diseases and external causes was not associated with hot or cold days [39]. Women, residents with low education, and those working in farms were the most vulnerable [38]. YLL (Years of Life Lost) increased with higher temperatures at shorter lags.

### Conclusion

This study clearly indicates the importance of the HDSS data for exploring weather/climate–disease and exposure–response relationships. There is very limited research on cause-specific mortality and extreme temperature in rural parts of India [39]. The research demonstrates the need for capacity building to improve surveillance and monitoring to detect changes in health that result from global climate change.

## Research infra structure building knowledge and capacity

It is primarily high-income countries (HICs) that, through emissions of greenhouse gases, have driven climate change, while the highest health damages have occurred and will continue to occur in LICs with the lowest adaptive capacity [2]. Almost 15 years ago, the Stern-report stated that ‘the poorest will be hit earliest and most severely’ [43]. Most publications about climate change and health originate from high-income countries; there have been significantly fewer studies from LMICs, resulting in a limited understanding of the risks. The 2020 Lancet Countdown on climate change and health reported that original research and evidence reviews on health and climate change increased by a factor of eight between the years 2007 and 2019, with the trend driven by researchers in HICs [1]. For example, the majority of the world’s research on heat waves and the effects of high temperatures on human life and health comes from HICs, while there is an under-representation of studies conducted in LMICs [44]. In 2009, Professor Peter Byass stated that physiological effects of heat might be one of the most important public health concerns to study in Africa [45].

**Figure 2.**
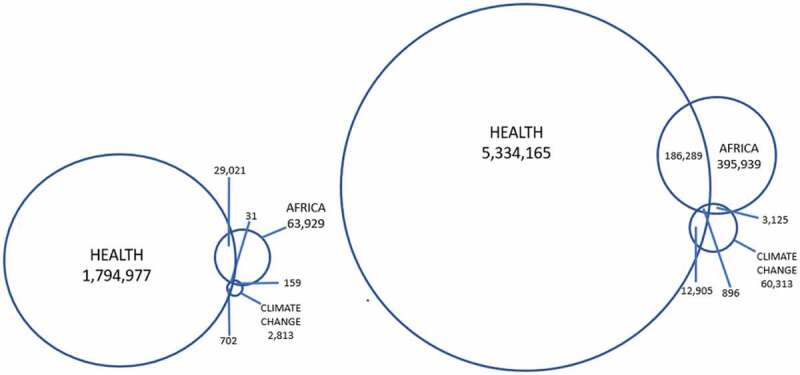
Results from a PubMed search for “health”, “Africa” and “climate change” June 26th 2009 to the left, and June 26th 2021 to the right, shows the intersections between the three terms. Total citations in 2009 was 1,891,632 and in 2021 5,790,417 (Based on Byass P, Climate change and population health in Africa: where are the scientist? [45]).

In the same year, Professor Byass searched the PubMed database using the keywords ‘health’, ‘Africa’, and ‘climate change’ [45]. The intersection between all three terms was only 31 (0.002%) out of almost 2 000 000 scientific citations that matched one or more of the terms. When a similar search was repeated on the same date 12 years later, in 2021, the total number of citations had increased to almost 5 800,000. The intersection between all three search terms had increased to 896 (0.02%), an increase that must be considered modest and the paucity of the intersection still striking. Just as Byass did then, one should reflect on what has, or has not, happened in terms of building scientific knowledge of population health and climate change in Africa during the last decade. Figure 2.

An obvious conclusion is that more epidemiological research output is needed from LMICs to increase resilience and decrease inequalities. Doing so requires building the capacity of local universities and researchers to collect, manage, analyse, and report on the data collected, and to use those data to develop, deploy, monitor, and evaluate interventions and adaptation options to inform local policy and decision makers. Research issues will not be solved by epidemiologists alone, but in the building of research capacity; researchers from different disciplines such as public health, meteorology, climate science, agriculture, remote sensing, and others need to be included [46]. Therefore, setting a nexus for HDSS-Climate-Capacity building is an innovation for leveraging research more aligned to and addressing key local scientific and health challenges. This infrastructure will enhance local ownership of activities, new opportunities for skills building, and development of local scientists and staff. The nexus will increase and improve research outputs including more first and last author papers. Recommendations drawn from regional/national-led studies may resonate more and lead to better uptake by local policymakers, providing more opportunities for senior local scientists/mentors to act as role models [47–49]. Local staff need to be trained in writing professional grant proposals that can be submitted to GCF or other funders [50,51]. Evidence drawn from climate-based HDSS will increase scientists’ understanding of the links between human health and climate change.

HDSSs have the potential to contribute important research outcomes, which may be particularly important in countries with no civil registration and vital statistics systems by collecting epidemiological data in defined populations on risks, exposures, and health outcomes [52]. Demographic surveillance minimally entails the longitudinal monitoring of the population in a geographically defined area, enumerating all individuals in the population, and obtaining basic information (i.e. age and sex).

The INDEPTH-network which is currently on hold due to governance disputes was comprised of 49 health and demographic surveillance sites (HDSS) that observed life events of over 3 000 800 people in 20 low- and middle-income countries in Africa, Asia, and Oceania [53]. Since its inception in 1998, the network gathered longitudinal population-based data records for all births, migrations, deaths, and causes of death, and on socioeconomic conditions via household interviews and through conducting verbal autopsies. It provided research infrastructure with the potential to contribute to important knowledge on health impacts from weather and climate variability and climate change.

Examples including demographic surveillance data from the INDEPTH network were used to quantify the risks of acute mortality from temperatures in LMICs. Underlying disease profiles and socio-economic determinants of health explained differences in effects in children, adults, and the elderly, especially where infectious disease mortality was high. In the Nouna HDSS in Burkina Faso, a short-term direct heat effect increased mortality, particularly in children below 5 years, while strong associations were found, especially in elderly, between rainfall events and daily mortality [54]. The availability and reliability of weather and climate data at research sites is critical for estimating weather and health impacts now and in the future [55]. Malaria research at INDEPTH-sites in Africa and Asia contributed to developing the scientific evidence base for prevention and control policies. Malaria incidence was monitored, particularly in children under five, and associations between climate variability and malaria transmission were studied in selected areas [56]. In 2014, data from several INDEPTH-member HDSS sites in Africa and Asia were used to assess malaria mortality, using verbal autopsy data. Rates were generally low in Asia in the 20-year period from 1992 to 2012 but were high in West Africa [57]. Kemri/CDC HDSS in Kenya found that malaria was associated with temperature and rainfall changes, with delays of up to 16 weeks. The results may be used for preparedness by predicting high transmission periods [58]. In 2012, a supplement of nine site-specific papers was published in Global Health Action. Publications examined weather conditions and population-level mortality and the situation from four African countries (Burkina Faso, Ghana, Kenya, and Tanzania) and two Asian countries (India and Bangladesh) offering regional perspectives for Asia, West-, and East Africa [59].

The African Population Cohort Consortium (APCC) is a new initiative with Wellcome Trust funding developing a new consortium for longitudinal population studies in the African continent to address knowledge gaps working across multiple domains such as human, environmental, socioeconomic, policy, and health systems [60]. Information is so far sparse, but the initiative aims to focus on strengthening African-led longitudinal population studies and discovery research to improve health and development in Africa.

Access to longitudinal data collected over decades is needed to provide robust estimates of the health impacts of climate change by ensuring that appropriate disease-, mortality-, and weather data are collected in a sufficiently large population. Combined with weather and climate data, exposure-response functions can be estimated, quantifying the current burden of climate-sensitive health outcomes and identifying which populations and regions are more vulnerable. Given the paucity of data from low-income settings, these quantifications are useful for increasing awareness of the health risks of climate change among policymakers and the public, and for informing development of health adaptation policies and programs to ensure that adaptation programs focus on protecting and promoting the health of the most vulnerable. One key adaptation is developing early warning and response systems that provide notice of environmental conditions that could lead to, for example, outbreaks of infectious diseases with sufficient time for health systems to take preventive actions. Another key adaptation is developing new or modifying current integrated surveillance programs to, for example, monitor changes in the geographic range or seasonality of infectious diseases.

Exposure-response functions are central for projecting how the burden of climate-sensitive health outcomes could change under a range of climate and development scenarios. These projections can inform national assessments, including the health contribution to Nationally Determined Contributions and to the Global Stocktake, and can be used to make an investment case for health adaptation. Further, understanding the magnitude and pattern of current impacts and potential risks would spur long-term monitoring to determine how vulnerability and risk change with additional climate change and spur evaluations of the effectiveness of adaptation programs. Knowledge gaps would highlight where additional data and analyses are needed. The HDSS infrastructure appears to be a unique opportunity for LMICs. Despite all its assets, there are still shortcomings that need to be addressed to make HDSS an ideal research tool for climate change and health. Indeed, morbidity data is not collected by most HDSS and high-resolution morbidity data are needed for accurate assessment of disease burden. There is also a lack of local weather station data that is more accurate for establishing cause and effect relationships, instead of modelled or proxy weather data. More effort should be concentrated on doing research highlighting the links between climate variability/change and morbidity in the population. Infrastructure is essential to locally collect weather data and to link them with HDSS morbidity data. There is a need for appropriate hardware and software for good-quality data collection and to perform data analyses. The capacity building of the team is essential: capacity is needed to set and maintain climate monitoring devices and equipment, and to master data processing and modelling skills. Promoting South–South and North–South cooperation can help fill the gaps in research infrastructure and capacity building. Finally, mentorship should be explored as a strategy to build capacity for knowledge translation research and practice for future research in the field of climate change and human health.

## Conclusions

There is limited research and many knowledge gaps about the associations between weather, climate variability, climate change and human health in sub-Saharan Africa and South Asia. Given the breadth of climate-sensitive health risks, the highlighted case studies focus on only a few of the outcomes that are likely already being affected by climate change. Future health risks will significantly affect the most vulnerable individuals, communities, and health systems. Because of the combined pressures of poverty, undernutrition, weak institutions, and overburdened public health infrastructure, populations in sub-Saharan Africa and South Asia may be more vulnerable to shifts in climate and extreme weather. Measures to protect human health are critical to reducing poverty and inequality at all scales.

More local research by local researchers is needed from LMICs related to weather, climate, and climate change. For example, conclusions about the health effects of heat waves, rising temperatures, and adaptation need to be drawn from research conducted locally in LMICs and not mainly based on research from HICs. To do this, research infrastructure and local research capacity need to be strengthened. Local researchers are generally best placed to understand local needs and vulnerabilities and they may have higher credibility in informing the public, policy-, and decisionmakers about effective and timely interventions. Through local researchers’ understanding of risks, vulnerabilities, and resources, the implementation of effective interventions can be made possible to a greater extent.

Achieving these goals requires building the capacity of local universities and researchers to design studies, collect, manage, analyse, and report the data collected. We assume efforts are underway to maintain the INDEPTH research infrastructure to enable the use of this treasure of data built over several decades in the network sites in Africa and Asia.

**The power of data – with reflections on capacity and equity**

BOX 1:

The highlighted case studies illustrate the benefits of capacity building among researchers in low- and middle-income countries to conduct analyses to inform local policy- and decision-makers. These researchers are best placed to understand the local vulnerabilities and capacities that interact with climate change to determine the magnitude and pattern of impacts [61]. In general, local analyses are more useful and have higher credibility for decision-makers when they are at relevant scales, considering local drivers of vulnerability. Incorporating understanding of the drivers of local risks into designing and implementing adaptation measures will increase their effectiveness and uptake and can address underlying inequities. The INDEPTH network provided an opportunity to build collaborations between researchers and local decision-makers to monitor and evaluate adaptations to identify best practices that could be shared across the sites to facilitate scaling up adaptation; a critically important task for these vulnerable locations as the climate continues to change.

BOX 2:

‘Tackling climate change could be the greatest global health opportunity of the 21st century’ [4].

Peter Byass was one of the Professors at the Umeå University (UmU) department of Epidemiology and global health whom in 2008 strongly supported the start and development of the research theme of climate change and health. Through the initiative, UmU at that time became one of the few universities with a focused research group that also engaged in capacity building by offering international postgraduate courses on the topic. Peter also particularly stressed researchers’ responsibility to translate knowledge into policy and practice. He sought broad international cross-disciplinary collaborations and had an advisory role in the Lancet Commission on Health and Climate Change published in 2015 and a leading role in the Lancet Countdown annual updates tracking progress on health and climate change. Peter was forward looking and combined his commitment for improved global health with the understanding of the threat climate change posed to global health and that the poorest would be hit earliest and most severely, often quoting the Stern-report [43]. His value-based compass was set to draw attention to the deep injustice in that the worst health damage from climate change would occur in low-income countries having the lowest capacity to adapt. He was committed to strengthening capacity, both human capacity and research capacity. The INDEPTH network was close to his heart, he was one of the founding contributors and the chair of INDEPTH’s scientific advisory committee for many years. He worked with the INDEPTH leadership to strengthen the capacity to develop and conduct research on weather, climate, and climate change in low-income countries in Africa and Asia. Peter was a warm-hearted colleague, a friend, and mentor for so many, which in his spirit will continue their committed work for equity in global health.

## Paper context

This paper gives an overview of the health impacts of weather, climate, and climate change, with a particular focus on vulnerable regions and populations in sub-Saharan Africa and South Asia. It discusses the importance of strengthening research infrastructure and local research capacity in LMICs related to weather, climate, and climate change to fill critical knowledge gaps, contribute to knowledge development, capacity building and designing interventions in countries where most needed. A conclusion is that local researchers are better placed to understand the local needs, risks, vulnerabilities, and resources, informing the public, policy-, and decisionmakers.
